# Biomechanical Effects of Distal Fragment Translation Guided by Target Postoperative Intermetatarsal Angle in Minimally Invasive Transverse Osteotomy for Hallux Valgus: A Patient-Specific Finite Element Study

**DOI:** 10.3390/bioengineering13101141

**Published:** 2026-09-30

**Authors:** Jiaming Shi, Yongze Jiang, Wanqi Xiong, Tao Zhang, Yujin Dong, Xinli Dong, Jiahe Liu, Baoyi Liu

**Affiliations:** 1 Department of Orthopedics, Affiliated Zhongshan Hospital of Dalian University, No. 6 Jiefang Street, Dalian 116001, Chinajiang20020219@163.com (Y.J.);; 2 The First Clinical College, Dalian Medical University, Dalian 116011, China

**Keywords:** hallux valgus, minimally invasive transverse osteotomy, intermetatarsal angle, distal fragment translation, finite element analysis, Kirschner wire

## Abstract

This study investigated the biomechanical effects of distal fragment translation guided by target postoperative first–second intermetatarsal angle (IMA) in minimally invasive transverse osteotomy (MITO) for hallux valgus using patient-specific finite element models. Three-dimensional models were reconstructed from computed tomography data of three female patients representing mild, moderate, and severe hallux valgus. The distal first-metatarsal fragment was translated laterally to achieve target postoperative IMA values of 9°, 8°, 7°, 6°, and 5°. Maximum von Mises stresses of the first through fifth metatarsals and Kirschner wires, together with mean osteotomy contact pressure and contact area, were evaluated under standardized static standing. As the target IMA decreased, the required translation increased, osteotomy contact area decreased, and mean contact pressure increased in all models. First-metatarsal stress increased most markedly in the severe model, reaching 25.74 MPa at 5°. In the mild and moderate models, further correction at the smaller IMA settings produced relatively limited additional changes in metatarsal stress while fixation stress and osteotomy-interface demands continued to increase. In contrast, the severe model showed continued increases in first-metatarsal and fixation stresses with further correction. These exploratory findings indicate that the mechanical effects of correction depend on both the target postoperative IMA and the distal fragment translation required to achieve it.

## 1. Introduction

Hallux valgus is a common forefoot deformity, with a pooled global prevalence of approximately 19%; it is more prevalent among women and older individuals [1]. It is a complex first-ray deformity involving malalignment of the first metatarsal, hallux, and sesamoid complex [2], and may lead to pain, impaired mobility and function, difficulty with footwear, and reduced quality of life [3]. In clinical practice, the hallux valgus angle (HVA) and first–second intermetatarsal angle (IMA), measured on weight-bearing radiographs, are widely used to assess deformity severity and the degree of surgical correction [4]. A recent systematic review also showed that the amount of correction that can be achieved is related to the severity of the preoperative angular deformity [5]. Thus, IMA is useful not only for describing first-ray alignment but also for assessing the extent of correction. 

Appropriate correction is an important part of hallux valgus surgical planning. Undercorrection may leave residual deformity and increase the risk of recurrence, and both preoperative and postoperative radiographic parameters have been associated with recurrence [6]. Excessive correction of bony or soft-tissue structures, on the other hand, may contribute to iatrogenic hallux varus [7]. First-ray malalignment can also alter forefoot loading and contribute to transfer metatarsalgia [8]. Importantly, satisfactory radiographic correction does not always restore normal forefoot loading. A systematic review and meta-analysis found that central metatarsal loading may remain elevated or even increase after surgery in some patients [9]. These findings suggest that a smaller postoperative angular value is not necessarily mechanically preferable. Finite element analysis (FEA) offers a way to examine internal loading patterns and compare the biomechanical consequences of different correction strategies [10].

Minimally invasive techniques are increasingly used for hallux valgus correction, and their clinical and radiographic outcomes are generally comparable to those of open procedures [11,12]. However, minimally invasive procedures differ considerably in osteotomy geometry, fragment manipulation, and fixation strategy. Distal transverse osteotomy of the first metatarsal allows substantial translational and rotational adjustment of the distal fragment. Yoon et al. described minimally invasive transverse distal metatarsal osteotomy (MITO), in which correction was achieved by lateral translation and rotational realignment of the distal fragment [13]. Recent clinical series have also shown that percutaneous distal transverse osteotomy can substantially improve HVA, IMA, and patient-reported outcomes [14,15]. Most existing studies, however, have focused on radiographic correction, clinical outcomes, and complications. Quantitative evidence remains limited regarding how much distal fragment translation is required to reach a specific postoperative IMA and how that translation affects metatarsal stress, Kirschner wire stress, and osteotomy contact mechanics. 

FEA has been increasingly used to investigate internal foot stresses, fixation loading, and osteotomy-site mechanics that are difficult to assess directly in vivo. A recent systematic review showed that hallux valgus FEA studies have mainly compared different osteotomy procedures, correction strategies, and fixation configurations [16]. Individual studies have examined distal-fragment displacement and metatarsal stress [17], osteotomy stability [18], fixation-related bone and implant stresses [19], and the effects of fragment translation on first-ray contact mechanics under simulated standing [20]. However, the relationship among target postoperative IMA, the translation required to achieve that target, and the resulting biomechanical response has not been well defined, particularly in patient-specific models evaluating these outcomes simultaneously. In this exploratory study, we developed three patient-specific finite element models representing mild, moderate, and severe hallux valgus. We calculated the lateral translation required to achieve postoperative IMA targets of 9°, 8°, 7°, 6°, and 5° and compared metatarsal stress, Kirschner wire stress, and osteotomy contact mechanics across these settings. The aim was to characterize model-specific relationships among target IMA, required distal fragment translation, and mechanical response, with the goal of providing biomechanical evidence for individualized correction planning. 

## 2. Materials and Methods

### 2.1. Study Design and Patients

This retrospective, patient-specific finite element study was approved by the Ethics Review Committee for Scientific Research Projects of the Affiliated Zhongshan Hospital of Dalian University (approval no. PJ-KY2026-047-1). Written informed consent was obtained from all patients for the use of their anonymized imaging data, and no additional imaging examinations were performed specifically for this study.

Three patients with complete preoperative imaging data were included to construct patient-specific finite element models representing mild, moderate, and severe hallux valgus. Each deformity category was represented by one model, and all three patients were female. Deformity severity was classified according to the hallux valgus angle (HVA) and first–second intermetatarsal angle (IMA) measured on preoperative weight-bearing radiographs, based on commonly adopted radiographic criteria [21]. Mild hallux valgus was defined as HVA < 20° and IMA < 13°, moderate hallux valgus as HVA 20–40° and IMA 13–16°, and severe hallux valgus as HVA > 40° and IMA > 16°. The mild model was based on a 48-year-old patient with left-sided hallux valgus (HVA 18°, IMA 11°), the moderate model on a 50-year-old patient with left-sided hallux valgus (HVA 30°, IMA 15°), and the severe model on a 53-year-old patient with right-sided hallux valgus (HVA 41°, IMA 17°).

Patients were eligible if they had a diagnosis of hallux valgus and available preoperative weight-bearing radiographs and foot CT data of sufficient quality for three-dimensional reconstruction. Exclusion criteria included a history of ipsilateral foot trauma or surgery, rheumatoid arthritis, gout, other conditions likely to substantially affect foot alignment or load distribution, or imaging data insufficient for reliable model reconstruction.

### 2.2. Finite Element Model Reconstruction

Foot CT images were obtained during previous clinical examinations using a 64-detector-row CT scanner (Siemens Healthineers, Erlangen, Germany). The original CT datasets were exported in Digital Imaging and Communications in Medicine (DICOM) format and imported into Mimics Research 21.0 (Materialise, Leuven, Belgium). Bony structures and the soft-tissue envelope were segmented using threshold-based segmentation, region growing, and manual editing to generate preliminary three-dimensional models. The reconstructed geometries were exported in stereolithography (STL) format and imported into Geomagic Wrap 2021 (3D Systems, Rock Hill, SC, USA) for surface repair, smoothing, and removal of redundant geometric features. The processed geometries were then imported into SOLIDWORKS 2024 (Dassault Systèmes, Vélizy-Villacoublay, France), where the bony structures were assembled and the articular cartilage geometries were constructed.

**Figure 1:**
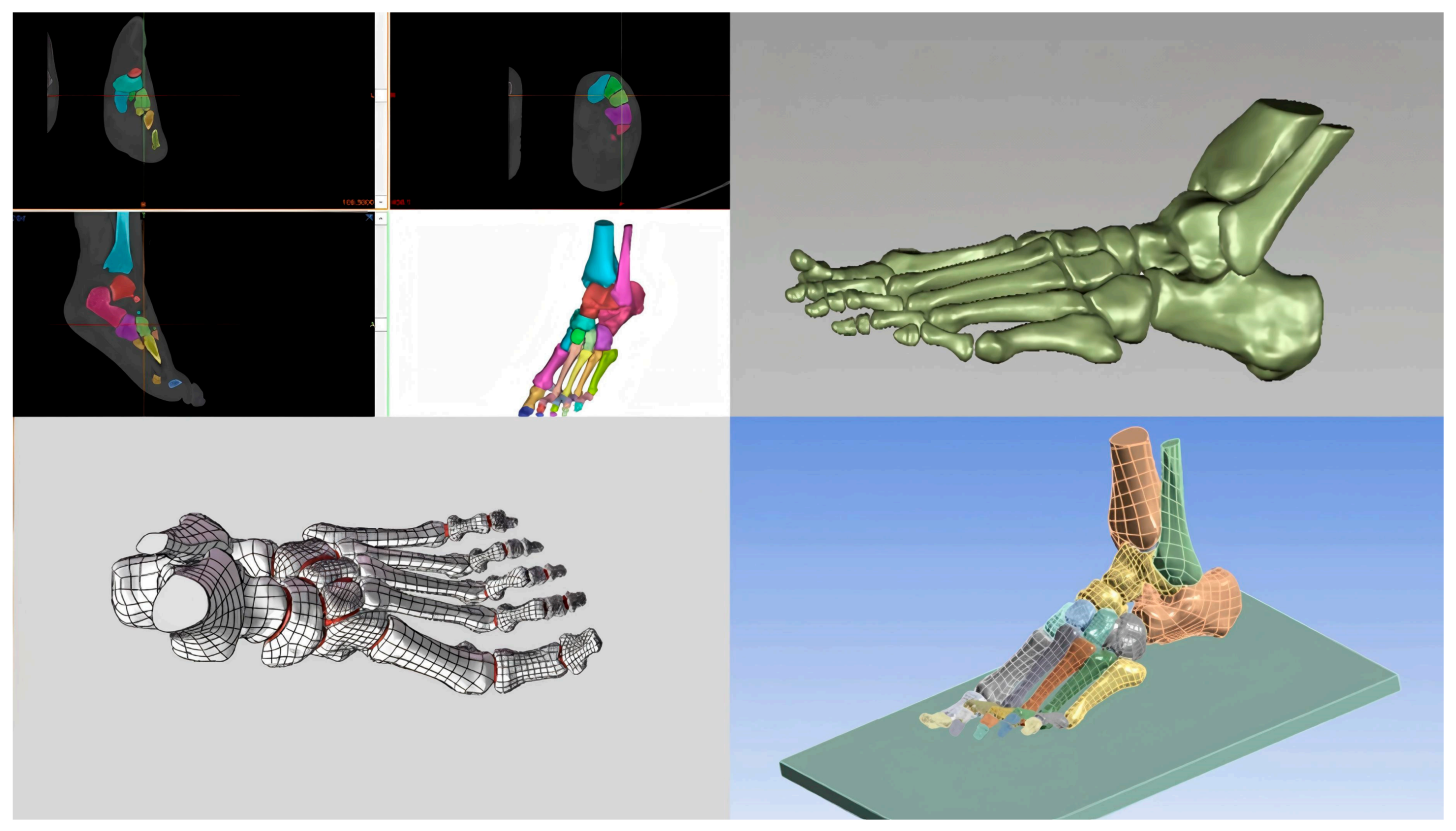
Workflow of patient-specific finite element model reconstruction. Different colors are used to distinguish anatomical structures and model components.

Each patient-specific foot model comprised 30 bony structures: the tibia, fibula, talus, calcaneus, cuboid, navicular, three cuneiforms, five metatarsals, two sesamoids, and 14 phalanges. The bony structures, articular cartilage, and soft-tissue envelope were assembled together with a ground plate, and the completed models were imported into ANSYS Workbench 2024 R1 (ANSYS Inc., Canonsburg, PA, USA) for FEA. The model reconstruction workflow is shown in Figure 1.

### 2.3. Mesh Generation and Convergence Analysis

All models were discretized using tetrahedral elements in ANSYS Workbench 2024 R1. The target element sizes were 4 mm for the soft-tissue envelope, 3 mm for the bony structures, 1.5 mm for the articular cartilage, and 0.5 mm for the Kirschner wires. The mild, moderate, and severe preoperative models contained 176,178, 199,017, and 163,038 elements and 315,081, 356,171, and 293,663 nodes, respectively.

Mesh convergence was evaluated using the moderate patient-specific postoperative model with an IMA of 7° as a representative configuration, including the osteotomy and Kirschner-wire fixation. With all other model settings unchanged, a finer mesh was generated, and three representative biomechanical outcomes were monitored: the maximum von Mises stress of the first metatarsal, the maximum von Mises stress of the Kirschner wires, and the mean contact pressure at the osteotomy interface. Further mesh refinement resulted in relative changes of 2.29%, 3.56%, and 2.68% in these three outcomes, respectively, all below the 5% convergence criterion adopted in a previous finite element study of the foot [22]. Therefore, the selected mesh was used for all subsequent simulations.

### 2.4. Material Properties and Contact Settings

The modeled tissues and fixation components were assigned homogeneous, isotropic, and linearly elastic material properties. Bone was represented as a single equivalent homogeneous material without separate modeling of cortical and cancellous components. This simplification was adopted to maintain consistent comparison of relative mechanical changes across different correction settings. The material properties adopted in the present models were based on previous finite element studies of the foot and hallux valgus [10,23] and are summarized in Table 1.

Articular cartilage was bonded to the underlying bone, while frictionless contact was defined between opposing cartilage surfaces to permit relative sliding. The bony structures were bonded to the surrounding soft-tissue envelope. The Kirschner wire–bone interfaces were modeled as bonded to represent an idealized firmly fixed postoperative condition without relative sliding or separation at the interface. This assumption was applied consistently across all postoperative configurations to standardize the fixation condition for comparative analysis. Frictional contact was defined between the soft-tissue envelope and the ground plate with a coefficient of friction of 0.60 [18]. Frictional contact was also assigned between the osteotomy surfaces, with a coefficient of friction of 0.46 based on previous experimental measurements of bone osteotomy interfaces [24].

### 2.5. Boundary Conditions and Loading

Static structural analyses were performed under standardized static standing conditions. Because the primary objective was to compare the mechanical effects of different correction settings across patient-specific geometries rather than the influence of individual body mass, a common reference body mass of 60 kg, corresponding to an approximate body weight of 600 N, was used to standardize the loading conditions across all three models. A vertical ground reaction force of 300 N, equivalent to approximately 50% of the reference body weight, was applied to the ground plate. The Achilles tendon force was set at 150 N, corresponding to 50% of the applied ground reaction force. This proportional loading scheme is consistent with previous finite element studies of the foot and hallux valgus, in which approximately half of body weight was applied as the ground reaction force and the Achilles tendon force was set at approximately 50% of the ground reaction force [10,23]. The same reference body mass, ground reaction force, and Achilles tendon force were applied to the mild, moderate, and severe patient-specific models and to all preoperative and postoperative configurations to enable direct comparison under identical loading conditions.

**Figure 2:**
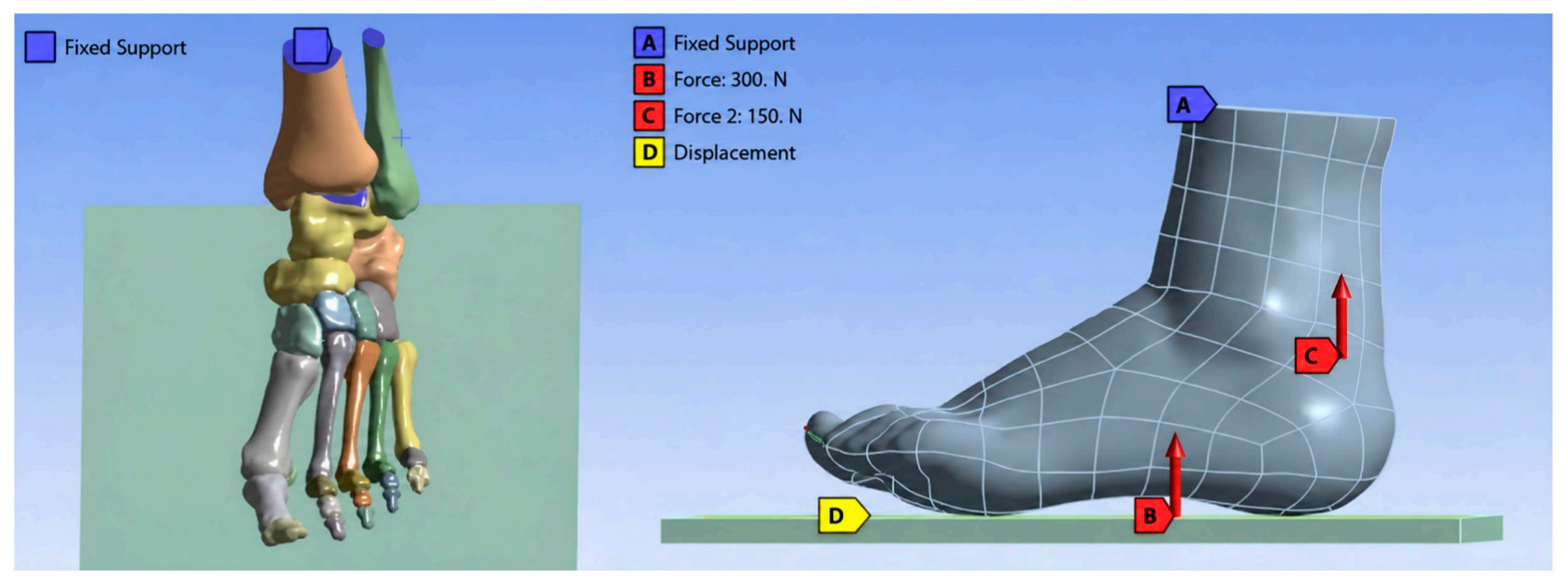
Boundary and loading conditions for the static standing simulation. Blue indicates fixed support, red arrows indicate the applied forces, and yellow indicates the displacement constraint.

The ground plate was constrained in the two horizontal directions while remaining free to translate vertically. The Achilles tendon force was applied vertically upward at its insertion on the calcaneus. The superior surfaces of the tibia, fibula, and talus, together with the proximal surface of the soft-tissue envelope, were fixed to provide a stable proximal reference and prevent rigid-body motion during static loading. Similar proximal constraints have been used in previous hallux valgus finite element studies [19,23]. The same boundary conditions were maintained across all patient-specific models and preoperative and postoperative configurations (Figure 2).

### 2.6. Model Plausibility Assessment

**Figure 3:**
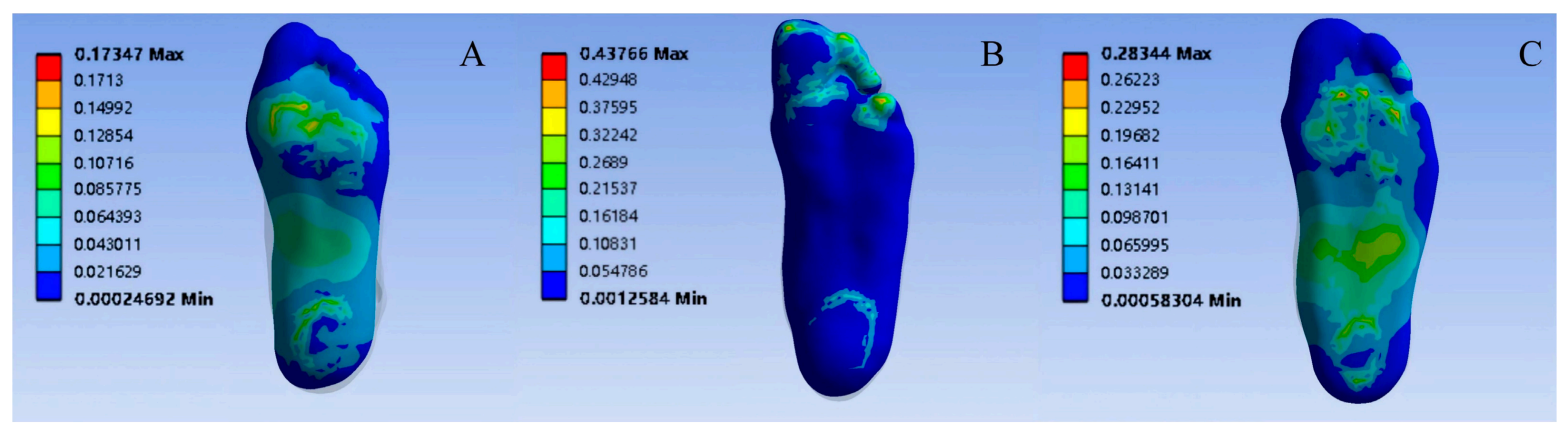
Preoperative plantar soft-tissue von Mises stress distributions under standardized static standing in the (**A**) mild, (**B**) moderate, and (**C**) severe models. Color scales are model-specific.

Because patient-specific plantar pressure measurements were unavailable, model plausibility was assessed by comparison with previously published hallux valgus finite element studies rather than by direct experimental validation. Under standardized static standing conditions, the preoperative models showed plantar soft-tissue von Mises stress concentrations mainly beneath the metatarsal heads and heel (Figure 3). Peak plantar soft-tissue von Mises stresses were 0.17, 0.44, and 0.28 MPa in the mild, moderate, and severe models, respectively. The overall plantar load-bearing pattern was broadly consistent with that reported in previous hallux valgus finite element studies [23,25], and the calculated stress magnitudes were of the same order of magnitude as plantar soft-tissue stresses reported under comparable static loading conditions [23]. This qualitative and order-of-magnitude agreement was considered to support the mechanical plausibility of the models for comparative analysis, but was not regarded as direct experimental validation.

### 2.7. Simulation of Minimally Invasive Transverse Osteotomy

First, the proximal phalanx of the hallux was rotated about the center of the first metatarsophalangeal joint to improve articular congruency with the first-metatarsal head, thereby approximating hallux realignment after lateral soft-tissue release. A transverse osteotomy was then created at the neck of the first metatarsal, with the osteotomy plane oriented perpendicular to the longitudinal axis of the metatarsal [13].

Five target postoperative IMA values were evaluated: 9°, 8°, 7°, 6°, and 5°. The 9° setting was included as a boundary value at the conventional radiographic cutoff for normal first–second intermetatarsal alignment (IMA < 9°), while values from 8° to 5° were included at 1° intervals to provide a predefined computational range representing progressively greater correction. Thus, the evaluated IMA settings were selected primarily for systematic biomechanical comparison rather than as clinically recommended postoperative targets. The preoperative IMA and first-metatarsal length (L) were measured on calibrated weight-bearing dorsoplantar radiographs. First-metatarsal length was defined as the distance from the metatarsal base to the center of the metatarsal head. The measured L values were 46.0, 51.0, and 41.8 mm for the mild, moderate, and severe models, respectively. The lateral translation required to achieve each target postoperative IMA was calculated using the planar geometric relationship described by Mavčič [26]. In this model, the correction angle (α − β), where α and β represent the preoperative and target postoperative IMA, respectively, forms a right triangle with the first-metatarsal length (L) as the adjacent side and the lateral translation of the metatarsal head (T) as the opposite side. Accordingly, T/L = tan(α − β), yielding T = L × tan(α − β). The distal fragment was then translated laterally along the osteotomy plane by the calculated distance. The translation distances for each target postoperative IMA are summarized in Table 2.

After translation, axial derotation of the distal fragment was performed about the longitudinal axis of the first metatarsal passing through the geometric center of the osteotomy surface. In the transverse plane, the angle between the medial–lateral axis of the osteotomy surface and a reference line parallel to the ground plate was measured. The distal fragment was then rotated by the same magnitude in the opposite direction until the two lines were approximately parallel. After translation and axial derotation, the postoperative IMA was remeasured on a standardized dorsoplantar projection of the three-dimensional model using the same metatarsal-axis definition as that used for the preoperative measurement. The remeasured postoperative IMA was then compared with the prespecified target value to verify that the intended angular correction had been achieved.

**Figure 4:**
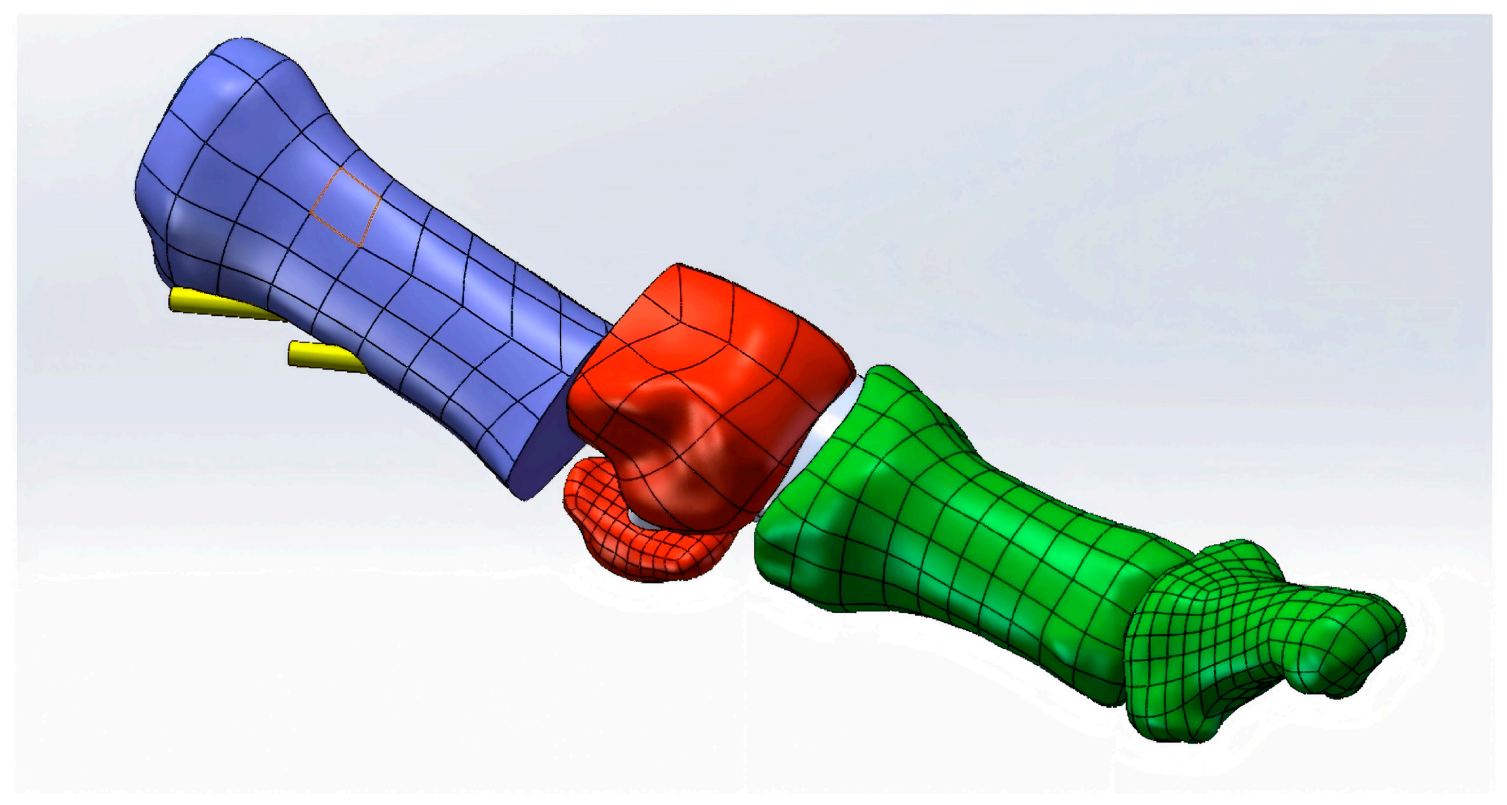
Representative postoperative first-ray model after simulated distal transverse osteotomy and double Kirschner wire fixation.

After verification of the postoperative IMA, the medial and lateral sesamoids were repositioned to approximate a normal positional relationship with the corrected first-metatarsal head. Thus, the postoperative reconstruction incorporated axial derotation of the distal first-metatarsal fragment and approximate repositioning of the sesamoids. However, first-metatarsal pronation, sesamoid alignment, and other components of three-dimensional first-ray alignment were not quantified or systematically varied as independent simulation parameters. Fixation was then simulated using two 2.0-mm-diameter stainless-steel Kirschner wires [19]. The first Kirschner wire was inserted through the medial cortex of the proximal first metatarsal, crossed the lateral cortex immediately proximal to the osteotomy, and engaged the lateral half of the translated metatarsal head. The second wire was positioned 10 mm distal and parallel to the first wire and engaged the central-to-lateral portion of the metatarsal head. Both wires terminated within the subchondral bone without penetrating the first metatarsophalangeal joint. The same entry points, inter-wire spacing, and distal target regions were maintained across all postoperative IMA configurations to ensure consistent comparison. A representative postoperative model is shown in Figure 4.

### 2.8. Outcome Measures

The finite element outcomes included the maximum von Mises stresses of the first through fifth metatarsals and the Kirschner wires, the mean contact pressure at the osteotomy interface, and the osteotomy contact area.

### 2.9. Data Analysis

Because each deformity category was represented by a single deterministic patient-specific finite element model, inferential statistical analyses were not performed. Mechanical outcomes were compared descriptively across the target postoperative IMA settings within each model, with emphasis on model-specific response patterns associated with increasing correction and distal fragment translation rather than on population-level inference. Kirschner wire stress values were interpreted comparatively across correction configurations; no material failure criterion or comparison with the yield or ultimate strength of the specific wire material was applied.

## 3. Results

### 3.1. Metatarsal Stress

The maximum von Mises stresses of the first through fifth metatarsals are summarized in Table 3, Table 4 and Table 5. Overall, the most evident stress changes occurred in the first and second metatarsals, whereas the third through fifth metatarsals showed relatively smaller variations.

In the mild model, first-metatarsal stress increased from 4.27 MPa preoperatively to 13.27 MPa at an IMA of 7°, with little further change at 6° and 5° (13.73 and 13.18 MPa, respectively). Second-metatarsal stress decreased from 8.75 MPa preoperatively to 5.14 MPa at 7°, followed by only small additional reductions to 5.03 and 4.93 MPa at 6° and 5°, respectively. Most of the stress changes in the first and second metatarsals therefore occurred as the IMA was reduced to approximately 7°, with comparatively limited additional changes at smaller IMA settings. The third through fifth metatarsals showed relatively small variations without a consistent monotonic trend.

In the moderate model, first-metatarsal stress increased from 2.30 MPa preoperatively to 14.44 MPa at an IMA of 6°, with minimal further change at 5° (14.52 MPa). Second-metatarsal stress decreased to 4.21 MPa at 6° and then increased slightly to 4.24 MPa at 5°. Similarly, third-metatarsal stress reached its minimum value at 6° (3.06 MPa) and increased slightly to 3.13 MPa at 5°. The fourth and fifth metatarsals showed only minor variations. Overall, stress changes in the first three metatarsals were comparatively limited between 6° and 5°.

In the severe model, first-metatarsal stress increased continuously from 6.36 MPa preoperatively to 25.74 MPa at an IMA of 5°, with no evident leveling-off across the evaluated range. In contrast, second-metatarsal stress decreased only slightly from 9.36 to 8.85 MPa, with minimal further change below 7°. Third-metatarsal stress decreased to 3.53 MPa at 7° but then increased to 3.72 and 4.21 MPa at 6° and 5°, respectively. Fourth-metatarsal stress showed a similar increase at the smaller IMA settings, whereas fifth-metatarsal stress remained nearly unchanged. Thus, reducing the IMA below approximately 7° was accompanied by continued increases in first-metatarsal stress and rising third- and fourth-metatarsal stresses, without the relative stabilization observed in the mild and moderate models.

**Figure 5:**
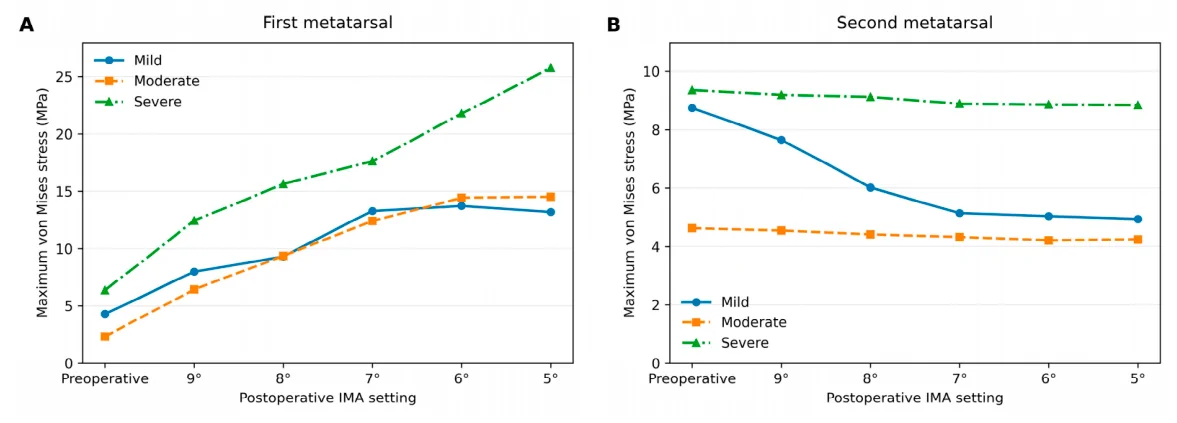
Maximum von Mises stresses of (**A**) the first metatarsal and (**B**) the second metatarsal under the preoperative condition and different postoperative IMA settings. The blue, orange, and green curves represent the patient-specific mild, moderate, and severe hallux valgus models, respectively.

The first and second metatarsals showed distinct response patterns across the three models. In the mild model, relatively limited additional changes were observed at IMA settings below approximately 7°, whereas the moderate model showed little further change between 6° and 5°. In contrast, the severe model showed a continued increase in first-metatarsal stress as the IMA decreased (Figure 5).

**Figure 6:**
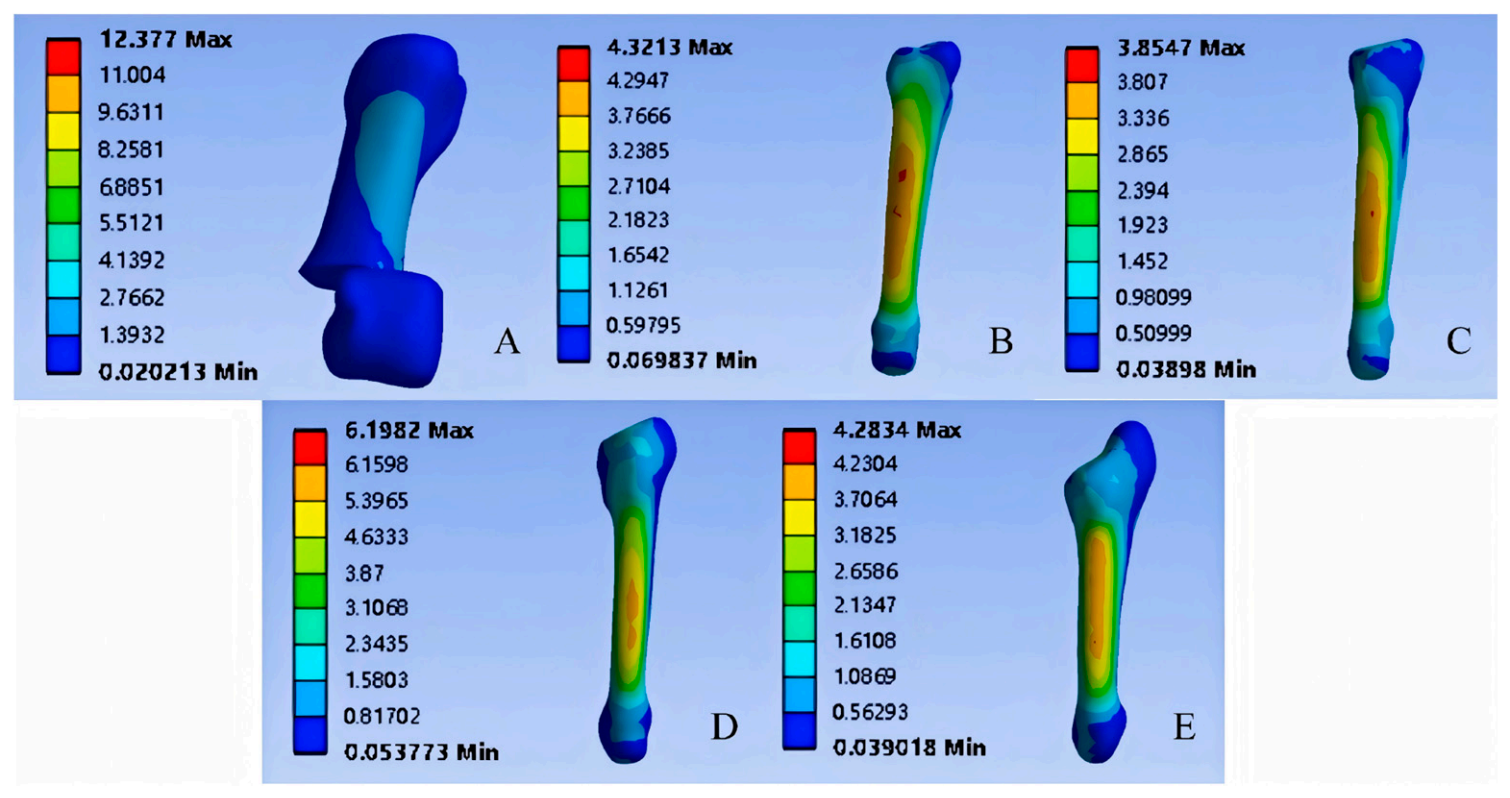
Representative von Mises stress distributions of the first through fifth metatarsals in the moderate model at a postoperative IMA of 7°. (**A**) First metatarsal; (**B**) second metatarsal; (**C**) third metatarsal; (**D**) fourth metatarsal; (**E**) fifth metatarsal. Color scales are panel-specific.

Representative stress contours for the moderate model at an IMA of 7° are shown in Figure 6. The maximum von Mises stresses of the first through fifth metatarsals were 12.38, 4.32, 3.85, 6.20, and 4.28 MPa, respectively. Stress in the first metatarsal was concentrated mainly near the osteotomy region, whereas stress concentrations in the second through fifth metatarsals were distributed predominantly along the metatarsal shafts. Panel-specific color scales were used to display the stress distribution within each metatarsal.

### 3.2. Kirschner Wire Stress

The maximum von Mises stresses of the Kirschner wires under different postoperative IMA settings are summarized in Table 6. Different stress-response patterns were observed among the three models.

In the mild model, Kirschner wire stress decreased as the IMA was reduced from 9° to 7°, reaching a minimum at 7°. Further reduction to 6° and 5° was associated with a marked increase in fixation stress. Thus, among the evaluated settings, the minimum Kirschner wire stress occurred at 7°, whereas smaller IMA settings were associated with increased simulated fixation stress.

In the moderate model, Kirschner wire stress increased progressively as the IMA decreased across the evaluated range. The largest increase between adjacent IMA settings occurred from 6° to 5°, indicating a more pronounced increase in simulated Kirschner wire stress at the smallest IMA setting.

In the severe model, Kirschner wire stress also increased progressively as the IMA decreased and exceeded that in the mild and moderate models at all postoperative IMA settings. The increase became more pronounced at the smaller IMA settings, particularly between 7° and 6° and between 6° and 5°. The maximum simulated Kirschner wire stress was 131.45 MPa at an IMA of 5°.

**Figure 7:**
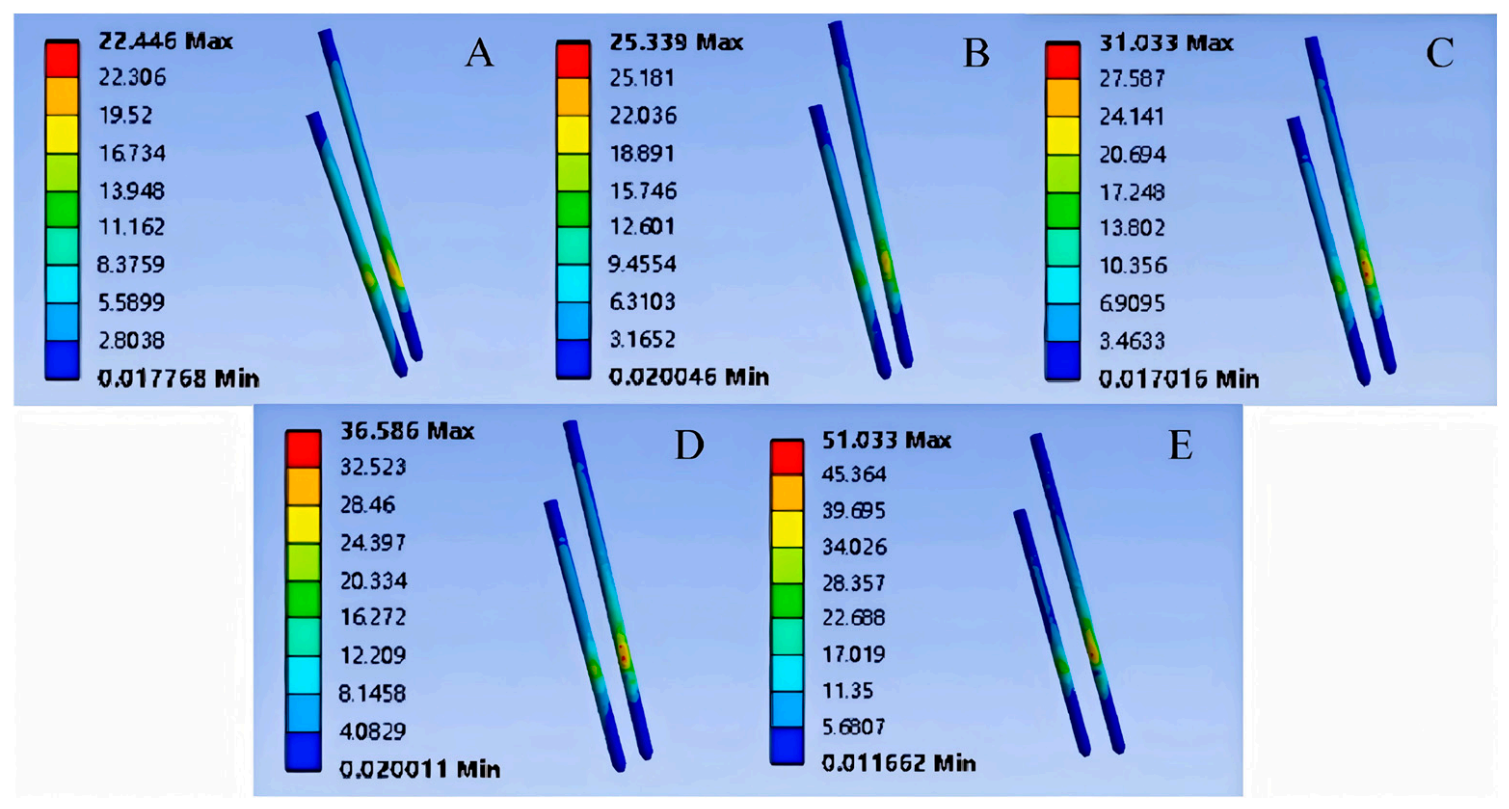
Representative von Mises stress distributions of the Kirschner wires in the moderate model under different postoperative IMA settings: (**A**) 9°; (**B**) 8°; (**C**) 7°; (**D**) 6° and (**E**) 5°. Color scales are panel-specific.

Representative Kirschner wire stress contours for the moderate model across different postoperative IMA settings are shown in Figure 7. Stress concentrations were located mainly in the distal intraosseous portions of the wires near the osteotomy region. As the IMA decreased from 9° to 5°, the location of the principal high-stress region remained generally similar, whereas the stress magnitude and local concentration progressively increased, with the greatest concentration observed at 5°. This spatial pattern was consistent with the progressive increase in maximum Kirschner wire stress shown in Table 6.

### 3.3. Osteotomy Contact Mechanics

The mean contact pressure and contact area at the osteotomy interface under different postoperative IMA settings are summarized in Table 7. In all three models, mean contact pressure increased and contact area decreased as the IMA was reduced, although the magnitude of these changes varied among the models.

In the mild model, mean contact pressure increased progressively from 0.31 MPa at an IMA of 9° to 0.51 MPa at 5°, while contact area decreased continuously from 152.44 to 109.80 mm^2^. These changes occurred gradually across the evaluated IMA range, with smaller IMA settings consistently associated with increased contact pressure and reduced osteotomy contact area.

In the moderate model, mean contact pressure increased from 0.31 MPa at 9° to 0.43 MPa at 6° and further to 0.58 MPa at 5°. At the same time, contact area decreased from 113.88 mm^2^ at 9° to 78.31 mm^2^ at 6° and 67.06 mm^2^ at 5°. The increase in mean contact pressure was most pronounced between 6° and 5°, while contact area continued to decline over the same interval.

In the severe model, mean contact pressure exceeded that in the mild and moderate models at all postoperative IMA settings. Mean contact pressure changed only slightly between 9° and 7° but increased more markedly as the IMA was further reduced to 6° and 5°. In contrast, contact area decreased continuously throughout the evaluated range. Changes in osteotomy contact mechanics therefore became more pronounced in the severe model at IMA settings below approximately 7°.

Overall, decreasing the postoperative IMA was associated with a progressive decrease in osteotomy contact area and an increase in mean contact pressure in all three models. These changes were relatively gradual in the mild model, became more pronounced between 6° and 5° in the moderate model, and were markedly greater below approximately 7° in the severe model.

## 4. Discussion

Although postoperative IMA is widely used to assess hallux valgus correction, quantitative evidence remains limited regarding the distal fragment translation required to achieve a given target IMA and its biomechanical consequences. Because radiographic correction does not necessarily correspond to normalized forefoot loading, FEA provides a useful means to examine internal mechanical responses that are difficult to assess in vivo [9,16,20]. Therefore, this exploratory study aimed to characterize the relationships among target postoperative IMA, required distal fragment translation, and biomechanical response across mild, moderate, and severe patient-specific models.

The main finding was that progressively smaller target IMA values required greater distal fragment translation and produced model-specific biomechanical trade-offs rather than a uniform mechanical response. In the mild and moderate models, further changes in metatarsal stress redistribution became relatively limited at the smaller IMA settings, whereas the mechanical demands on the fixation construct and osteotomy interface continued to increase. In contrast, the severe model showed no comparable relative stabilization, with further correction accompanied by continued increases in first-metatarsal and fixation stresses, together with progressively reduced osteotomy contact area and increased contact pressure. These findings suggest that postoperative IMA should not be considered in isolation; the translation required to achieve a given target and the associated mechanical consequences should also be considered during correction planning. Because each deformity category was represented by a single patient-specific model, these results should be interpreted as exploratory, model-specific trends rather than universal severity-specific target values. 

In the present models, first-metatarsal stress generally increased after correction, whereas second-metatarsal stress decreased, indicating a redistribution of internal loading between the first ray and the central forefoot. These osseous von Mises stresses should not be interpreted as direct surrogates for plantar pressure, but they provide complementary information on internal mechanical changes. Clinical pedographic studies have reported variable postoperative loading responses, including increased plantar pressure beneath the first metatarsal head [27], reduced pressure beneath the second metatarsal head after combined hallux valgus and lesser-ray surgery [28], and persistent central metatarsal loading despite substantial radiographic correction after MICA [29]. Together, these findings indicate that satisfactory radiographic correction does not necessarily produce a uniform change in forefoot loading. Accordingly, the stress changes observed in the present models are better interpreted as model-specific internal mechanical redistribution rather than normalization of plantar loading. In addition, in the mild and moderate models, further changes in metatarsal stress became relatively limited at the smaller IMA settings, suggesting that greater geometric correction does not necessarily lead to proportionally greater changes in metatarsal load distribution. 

In addition to metatarsal load redistribution, increasing correction was associated with changes in the simulated stress response of the fixation construct and the contact mechanics of the osteotomy interface. With the fixation configuration and loading conditions held constant, Kirschner wire stress tended to increase at smaller IMA settings, indicating an increase in simulated fixation stress as distal fragment translation increased. The maximum simulated Kirschner wire stress was 131.45 MPa in the severe model at an IMA of 5°. Because no material failure criterion or verified yield or ultimate strength for the specific Kirschner-wire material was incorporated into the model, this value should be interpreted as a model-derived stress response rather than evidence of wire yielding, fracture, or mechanical failure. Previous finite element research has likewise demonstrated local stress concentration in Kirschner wire fixation after minimally invasive hallux valgus osteotomy [30]. At the osteotomy interface, decreasing IMA was accompanied by a progressive reduction in contact area and an increase in mean contact pressure. These two parameters should be considered together, because their concurrent changes indicate that interface loading became concentrated over a smaller effective bony contact area as translation increased. Previous finite element analysis has associated larger osteotomy contact area with less separation at the osteotomy site and more favorable mechanical stability [31], while patient-specific three-dimensional modeling has shown that surgical geometry and translation strategy can substantially affect osteotomy contact area [32]. Taken together, these findings indicate that greater correction, particularly at smaller IMA settings, may impose increased local mechanical demands on both the fixation construct and the osteotomy interface. These contact-mechanical findings are best interpreted as descriptors of the simulated immediate postoperative mechanical environment, while their relationship to bone healing, nonunion, and other clinical outcomes remains to be established.

Considering the combined changes in metatarsal stress, Kirschner wire stress, and osteotomy contact mechanics, a model-specific trade-off was observed between further correction and increasing local mechanical demands. In the mild model, once the IMA reached approximately 7–8°, further correction produced relatively limited additional changes in metatarsal stress, whereas fixation and osteotomy-interface demands continued to increase. A similar pattern was observed in the moderate model at approximately 6–7°. These IMA ranges also fell within the commonly accepted radiographic normal range (<9°) [33]. Clinical studies have shown that greater lateral translation can result in greater IMA correction [34], although the amount of IMA reduction cannot be determined by translation alone [35]. In addition, three-dimensional model-assisted planning has been reported to improve control of first-metatarsal head lateralization and IMA correction [36]. Accordingly, relative stabilization of metatarsal stress was observed around 7–8° in the mild model and 6–7° in the moderate model, while fixation and osteotomy-interface demands continued to increase with further correction. These model-specific patterns should not be interpreted as clinically optimal postoperative IMA ranges. These findings further illustrate that the mechanical response is influenced by both the target postoperative IMA and the translation required to achieve it.

Unlike the mild and moderate models, the severe model showed no comparable relative stabilization across the evaluated IMA range, and further correction remained associated with increasing mechanical demands on the fixation construct and osteotomy interface. This does not imply that distal minimally invasive osteotomy is unsuitable for severe hallux valgus; rather, it suggests that postoperative IMA alone may be insufficient to guide correction when a larger deformity must be addressed. Within the geometric framework of the present study, the translation required to achieve a given target IMA depends on the preoperative IMA and patient-specific first-metatarsal geometry. As a result, the same postoperative IMA may require different amounts of translation and lead to different mechanical consequences among patients. Current clinical guidance also emphasizes individualized planning for severe hallux valgus [37]. Moreover, hallux valgus is a three-dimensional deformity, and factors such as first-metatarsal pronation, sesamoid position, and overall first-ray alignment may influence the correction strategy [38,39]. Clinical studies have shown that percutaneous distal osteotomy can achieve substantial correction in severe hallux valgus [40], while different procedures may achieve similar angular correction despite differences in residual first-metatarsal pronation [41]. Therefore, correction planning in severe deformity should consider the target IMA together with the translation required to achieve it and other patient-specific three-dimensional characteristics, rather than treating a smaller postoperative IMA as the sole objective.

This study has several limitations. First, only three patient-specific finite element models were included, with one patient representing each deformity grade, and all three patients were female. Therefore, the observed differences and response patterns should be regarded as exploratory and model-specific rather than as general severity-dependent relationships. Although axial derotation and approximate sesamoid repositioning were incorporated into the postoperative models, first-metatarsal pronation, sesamoid alignment, and other aspects of three-dimensional first-ray alignment were not quantified or systematically varied; therefore, their independent mechanical effects could not be evaluated. Second, several modeling simplifications were adopted. The tissues were assumed to be homogeneous, isotropic, and linearly elastic; foot ligaments and muscle forces of smaller magnitude were not explicitly modeled; and the Kirschner wire–bone interface was assumed to be fully bonded, thereby excluding possible micromotion or sliding. Together with the fixed proximal boundary, these idealized assumptions may influence the absolute load-transfer and local stress magnitudes; therefore, the predicted fixation stresses should be interpreted primarily as comparative mechanical responses across configurations rather than as direct predictions of interface behavior. In addition, standardized loading based on a reference body mass of 60 kg was applied to all models rather than patient-specific body-weight loading. Third, all analyses were performed under static standing conditions and therefore did not reproduce the dynamic muscle forces, joint loads, and plantar contact conditions that occur during gait. Finally, the models primarily represent the immediate postoperative mechanical environment and do not account for bone healing or remodeling. Independent experimental and postoperative clinical validation were also unavailable, and the relationship between the simulated mechanical findings and clinical outcomes remains to be established. Future studies incorporating larger patient-specific cohorts, more physiologic modeling, dynamic loading, and clinical follow-up are warranted.

## 5. Conclusions

Patient-specific FEA demonstrated that the mechanical effects of correction depended on both the target postoperative IMA and the distal fragment translation required to achieve it. Across the three computational models, progressively greater correction produced distinct model-specific changes in metatarsal stress, fixation stress, and osteotomy contact mechanics. Given that each deformity category was represented by a single patient-specific model, these findings should be interpreted as exploratory biomechanical patterns rather than as severity-specific clinical target ranges.

## Figures and Tables

**Table 1:** Material properties used in the finite element models.

Component	Elastic Modulus (MPa)	Poisson’s Ratio
Equivalent homogeneous bone	7300	0.30
Kirschner wire	200,000	0.30
Articular cartilage	10	0.40
Soft-tissue envelope	1.15	0.49
Ground plate	17,000	0.40

**Table 2:** Calculated lateral translation distances (mm) for different target postoperative IMA values.

Model	Preoperative IMA (°)	IMA 9°	IMA 8°	IMA 7°	IMA 6°	IMA 5°
Mild	11	1.6	2.4	3.2	4.0	4.8
Moderate	15	5.4	6.3	7.2	8.1	9.0
Severe	17	5.9	6.6	7.4	8.1	8.9

Note: IMA, first–second intermetatarsal angle. Translation distances were rounded to the nearest 0.1 mm.

**Table 3:** Maximum von Mises stresses of the first through fifth metatarsals in the mild model (MPa).

Metatarsal	Preoperative	IMA 9°	IMA 8°	IMA 7°	IMA 6°	IMA 5°
1st metatarsal	4.27	7.98	9.32	13.27	13.73	13.18
2nd metatarsal	8.75	7.65	6.02	5.14	5.03	4.93
3rd metatarsal	6.52	6.37	6.37	6.04	6.41	6.70
4th metatarsal	6.96	6.94	7.00	6.90	6.96	6.79
5th metatarsal	5.05	4.94	4.95	5.00	4.97	4.95

**Table 4:** Maximum von Mises stresses of the first through fifth metatarsals in the moderate model (MPa).

Metatarsal	Preoperative	IMA 9°	IMA 8°	IMA 7°	IMA 6°	IMA 5°
1st metatarsal	2.30	6.42	9.38	12.38	14.44	14.52
2nd metatarsal	4.62	4.54	4.41	4.32	4.21	4.24
3rd metatarsal	4.25	4.21	3.96	3.85	3.06	3.13
4th metatarsal	6.29	6.23	6.21	6.20	6.11	6.15
5th metatarsal	4.37	4.33	4.31	4.28	4.25	4.24

**Table 5:** Maximum von Mises stresses of the first through fifth metatarsals in the severe model (MPa).

Metatarsal	Preoperative	IMA 9°	IMA 8°	IMA 7°	IMA 6°	IMA 5°
1st metatarsal	6.36	12.42	15.63	17.58	21.78	25.74
2nd metatarsal	9.36	9.20	9.13	8.90	8.87	8.85
3rd metatarsal	3.77	3.72	3.70	3.53	3.72	4.21
4th metatarsal	6.43	6.30	6.16	6.12	6.66	7.04
5th metatarsal	2.97	2.96	2.95	2.94	2.93	2.92

**Table 6:** Maximum von Mises stresses of the Kirschner wires under different postoperative IMA settings (MPa).

Model	IMA 9°	IMA 8°	IMA 7°	IMA 6°	IMA 5°
Mild	72.56	67.12	66.82	82.36	84.47
Moderate	22.45	25.34	31.03	36.59	51.03
Severe	93.00	97.33	101.85	112.36	131.45

**Table 7:** Mean contact pressure and contact area at the osteotomy interface under different postoperative IMA settings.

IMA (°)	Mild		Moderate		Severe	
	P (MPa)	A (mm^2^)	P (MPa)	A (mm^2^)	P (MPa)	A (mm^2^)
9	0.31	152.44	0.31	113.88	0.75	63.73
8	0.38	141.63	0.36	101.80	0.77	56.93
7	0.40	130.90	0.38	89.91	0.78	49.31
6	0.46	120.28	0.43	78.31	1.02	42.83
5	0.51	109.80	0.58	67.06	1.22	35.68

Note: P = mean contact pressure; A = contact area; IMA = first–second intermetatarsal angle.

## Data Availability

The data supporting the findings of this study are available from the corresponding author upon reasonable request, subject to applicable ethical and privacy considerations.
